# SH_2_-Inositol Phosphatase 1 Negatively Influences Early Megakaryocyte Progenitors

**DOI:** 10.1371/journal.pone.0003565

**Published:** 2008-10-29

**Authors:** Lia E. Perez, Caroline Desponts, Nancy Parquet, William G. Kerr

**Affiliations:** 1 Immunology Program, H. Lee Moffitt Comprehensive Cancer Center and Research Institute, Tampa, Florida, United States of America; 2 Blood and Marrow Transplantation Program, H. Lee Moffitt Comprehensive Cancer Center and Research Institute, Tampa, Florida, United States of America; 3 Department of Chemistry, The Scripps Research Institute, San Diego, California, United States of America; Cleveland Clinic, United States of America

## Abstract

**Background:**

The SH2-containing-5′inositol phosphatase-1 (SHIP) influences signals downstream of cytokine/chemokine receptors that play a role in megakaryocytopoiesis, including thrombopoietin, stromal-cell-derived-Factor-1/CXCL-12 and interleukin-3. We hypothesize that SHIP might control megakaryocytopoiesis through effects on proliferation of megakaryocyte progenitors (MKP) and megakaryocytes (MK).

**Methodology and Principal Findings:**

Herein, we report the megakaryocytic phenotype and MK functional assays of hematopoietic organs of two strains of SHIP deficient mice with deletion of the SHIP promoter/first exon or the inositol phosphatase domain. Both SHIP deficient strains exhibit a profound increase in MKP numbers in bone marrow (BM), spleen and blood as analyzed by flow cytometry (Lin^−^c-Kit^+^CD41^+^) and functional assays (CFU-MK). SHIP deficient MKP display increased phosphorylation of Signal Transducers and Activators of Transcription 3 (STAT-3), protein kinase B (PKB/AKT) and extracellular signal-regulated kinases (ERKs). Despite increased MKP content, total body number of mature MK (Lin^−^c-kit^−^CD41^+^) are not significantly changed as SHIP deficient BM contains reduced MK while spleen MK numbers are increased. Reduction of CXCR-4 expression in SHIP deficient MK may influence MK localization to the spleen instead of the BM. Endomitosis, process involved in MK maturation, was preserved in SHIP deficient MK. Circulating platelets and red blood cells are also reduced in SHIP deficient mice.

**Conclusions/Significance:**

SHIP may play an important role in regulation of essential signaling pathways that control early megakaryocytopoiesis *in vivo*.

## Introduction

SH2-containing-5′inositol phosphatase-1 (SHIP) can catalyze the removal of the 5′ phosphate group from phosphatidylinositol-3,4,5-phosphate (PIP3) and inositol-1,3,4,5–tetrakisphosphate (IP4) [1], [2]. In this manner, SHIP can prevent the recruitment of pleckstrin homology containing proteins to the plasma membrane or prevent calcium uptake and thus regulate survival, proliferation and activation of hematopoietic cells [3], [4]. SHIP deficiency has profound functional and pathological consequences as SHIP mutant mice suffer from osteoporosis [5], and a myeloproliferative disorder that leads to lung consolidation [6]. Analysis of SHIP mutant mice has demonstrated a pivotal role for SHIP *in vivo* in regulating the homeostasis of various cell types including tissue macrophages, osteoclasts, myeloid, natural killer (NK) and hematopoietic stem cells (HSC) [4]–[7]. We have shown that despite expansion of the HSC compartment, SHIP deficient mice have reduced long-term engraftment capacity and have an altered homing capacity due to reductions in key chemotaxis and adhesion receptors [8]. Furthermore, SHIP deficient mice have a promiscuous NK cell repertoire that permits engraftment of completely mismatched bone marrow (BM) without graft-versus-host disease [7].

SHIP is known to influence signaling pathways downstream of receptors for chemokines and cytokines involved in megakaryocytopoiesis and thrombopoiesis, such as thrombopoietin (TPO) [9]–[12], Stromal-cell-derived-Factor 1 (SDF-1/CXCL-12), [13]–[16] and interleukin (IL)-3 [17]. TPO influences megakaryocyte (MK) development by controlling proliferation, differentiation, survival and endoduplication [18]. Circulating platelets sequester free TPO, and thereby limit megakaryocytopoiesis during steady-state hematopoiesis [19]–[23]. Upon binding of TPO to its receptor, *c-mpl*, phosphatidylinositol 3-kinase (PI3K) and mitogen-activated protein kinase (MAPK) pathways are activated promoting cycling and survival of MK [9], [24], [25]. TPO binding to *c-mpl* also leads to SHIP phosphorylation [9], which may be a negative regulator of these signaling pathways. Furthermore, Lyn kinase deficient mice have an increased MK progenitor pool associated with a reduction of SHIP phosphorylation, implicating SHIP in MK development inhibition [26].

SDF-1/CXCL12 induces transendothelial MK migration and platelet production *in vitro* {Hamada, *et al* 1998, Wang, *et al* 1998} and increases platelets in NOD/SCID and Balb/c mice [15], [27]. We have shown that SDF-1 enhances human thrombopoiesis in xenotransplanted NOD/SCID mice [28]. SDF-1 and fibroblast growth factor-4 allow hematopoietic progenitors to relocate to a BM microenvironment that is permissive and instructive for MK maturation and thrombopoiesis [29]. SHIP deficient myeloid progenitors exhibit enhanced chemotaxis towards SDF-1/CXCL-12, indicating that SHIP influences signaling downstream of its receptor, CXCR-4 [30]. However, our recent demonstration that SHIP deficiency reduces both the surface density of CXCR-4 and homing of HSC indicates SHIP deficiency can also compromise CXCR-4 signaling [8].

It has been reported that SHIP deficient BM have decreased number of colony-forming-unit megakaryocytes (CFU-Mk) [31], and SHIP has also been shown to regulate PIP3 levels after thrombin or collagen induced platelet activation [32], [33]. Based on the defined role for SHIP in signaling pathways for cytokine/chemokines that also regulate MK and platelet biology, we hypothesized that SHIP might be involved in the regulation of megakaryocytopoiesis and platelet production *in vivo*. Herein, we report that two strains of SHIP deficient mice exhibit increased numbers of megakaryocyte progenitors (MKP) in hematopoietic organs as determined by flow cytometry (Lin^−^c-Kit^+^CD41^+^) and functional assays (colony forming unit (CFU)-MK). Despite the increase in MKP, mature MK (Lin^−^c-Kit^−^CD41^+^) numbers are not significantly changed since MK redistribute to other organs in the SHIP deficient animal. Moreover, MK endoduplication function is preserved in SHIP deficient MK, as well as circulating platelet numbers, which are not proportionally increased as would be expected. Interestingly, SHIP deficient MKP exhibit increased phosphorylation of Signal Transducers and Activators of Transcription 3 (STAT-3), protein kinase B (PKB/AKT) and extracellular signal-regulated kinases (ERKs). Furthermore, CXCR-4 surface expression is reduced on SHIP deficient MK as we previously showed for HSC [8] contributing, in part, to localization of MK progenitors to peripheral hematopoietic organs. These findings suggest that SHIP might be important for the control of early MK development.

## Results

### SHIP deficient bone marrow, spleen and blood contain increased numbers of functional MKP

**Figure 1:**
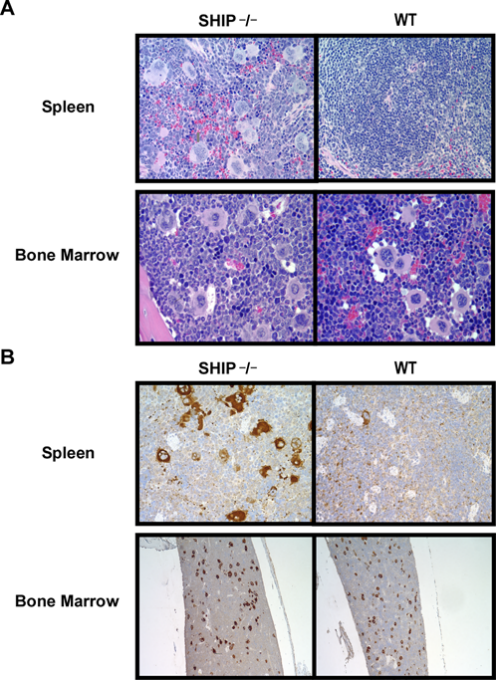
MK morphology and Glycoprotein IIB-IIIa (CD41) immunostain in BM and spleen of SHIP deficient mice. (A). Hematoxylin-Eosin staining of BM and spleen of SHIP^−/−^ (−/−) and wild type (WT) littermate controls. Spleen of SHIP deficient mice is characterized by a massive infiltrate with morphologically appearing MK (left panel) compared to WT littermates (right panel). BM of SHIP^−/−^ mice contained hypolobulated micro-MK compared to hyperlobulated MK in WT BM. Magnification: ×60. Results are representative of those obtained in 3 animals per experimental group (B). Glycoprotein IIB-IIIA (CD41) immuno-histochemistry. Spleen of SHIP^−/−^ mice contained a higher percentage of CD41^+^ cells compared to WT spleen. Magnification: ×40. BM of SHIP deficient mice contained comparable number of CD41^+^ cells compared to WT. Magnification: ×60.

Histological evaluation of Hematoxylin-Eosin stained spleens from two strains of SHIP deficient mice revealed that there is an impressive infiltrate of morphologically appearing MK. BM histopathology revealed that MK in SHIP deficient (SHIP^−/−^) BM are equally represented compared to wild type (WT) control. MK morphology in SHIP deficient mice showed that they are hypolobulated micro-MK when compared to WT BM which contained hyperlobulated MK (Figure 1A). Immuno-staining with anti-Glycoprotein IIB-IIIA (CD41a) antibody corroborated the MK lineage nature of the cells observed in spleen and BM (Figure 1B).

**Figure 2:**
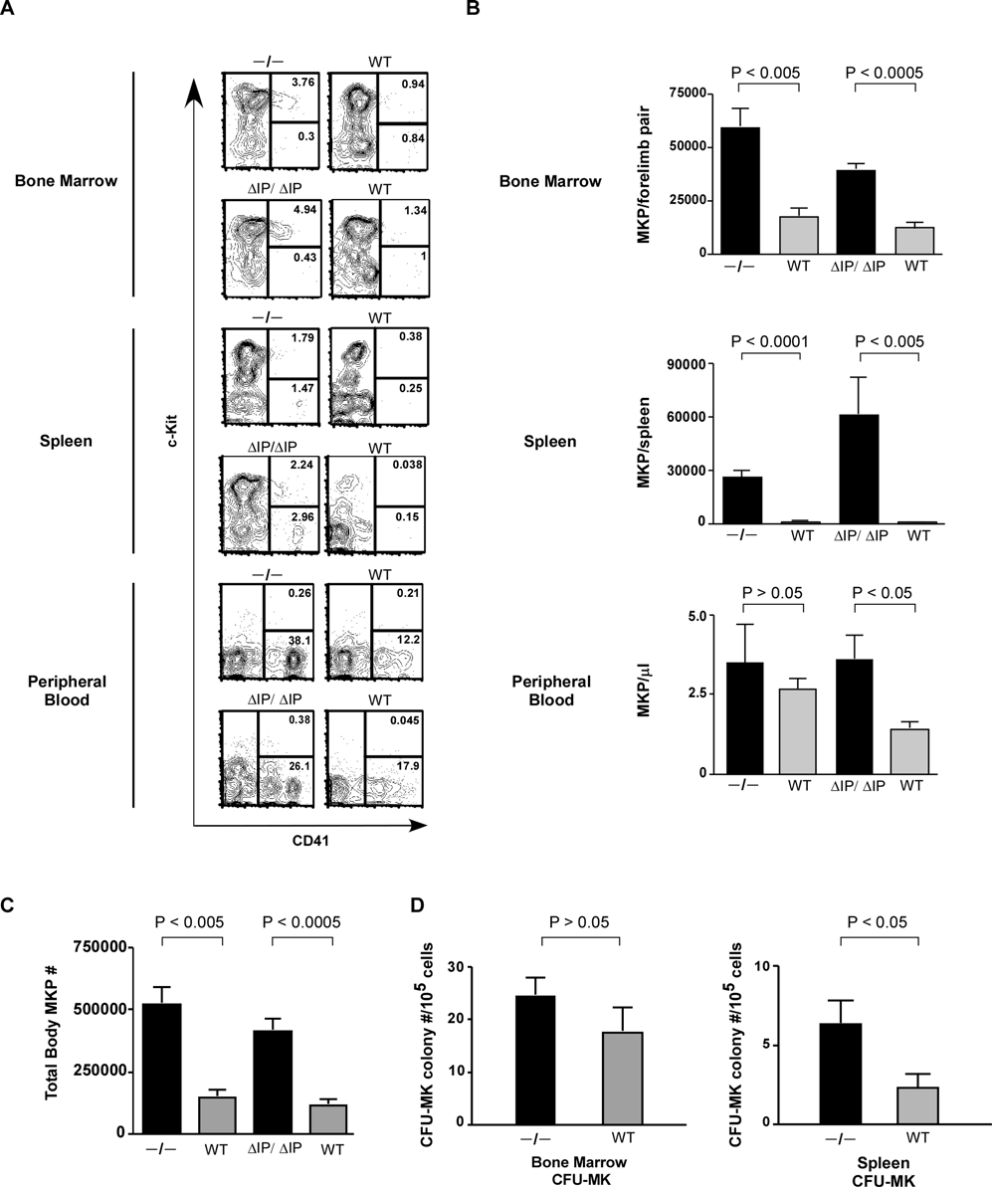
SHIP deficient mice have increased number of functional MKP in hematopoietic organs. (A) BM, spleen, and PB MKP phenotype in SHIP^−/−^ (−/−) and SHIP^ΔIP/ΔIP^ (ΔIP/ΔIP) mice. Shown are representative flow cytometry dot plots of CD41 (*x*-axis) versus c-Kit (*y*-axis) after gating on live and Lin^−^ cells. MKP (Lin^−^c-Kit^+^CD41^+^) are represented in the right upper quadrants and MK (Lin^−^c-Kit^−^CD41^+^) are represented in the right lower quadrants. Percentages for each population are indicated on each plot. (B) Represented are the mean±standard error mean (SEM) (n = 4 for experimental groups WT C57Bl6 and SHIP^−/−^, while n = 6 for experimental groups WT 129SvJ and SHIP^ΔIP/ΔIP^) of the absolute numbers of MKP in BM, spleen and PB of SHIP deficient mice (black columns) compared to respective WT littermates (gray columns). Mice strains specified in the graphs. (C) Represented is the mean±SEM (n = 4 for experimental groups WT C57Bl6 and SHIP^−/−^, while n = 6 for experimental groups WT 129SvJ and SHIP^ΔIP/ΔIP^) of total body MKP numbers determined as the calculated sum of MKP in one whole spleen plus MKP in one femur ×16.6 (since one femur is estimated to contained 6% the total marrow) [3]. (D) Represented is the mean±SEM of CFU-MK colonies in BM and spleen of SHIP^−/−^ (n = 7) and WT (n = 7) littermates. Statistical analysis was established using the Mann-Whitney test. *p* values indicated on each graph.

To further characterize the megakaryocytic compartment in SHIP deficient mice, we assessed the presence of early MKP, immuno-stained as Lin^−^c-Kit^+^CD41^+^, and of more differentiated MK, identified as Lin^−^c-Kit^−^ CD41^+^ (Figure 2A) [34]. The MKP (Lin^−^c-Kit^+^CD41^+^) compartment in the BM, spleen and peripheral blood (PB) of both SHIP deficient mice (SHIP^−/−^ and SHIP^ΔIP/ΔIP^) was increased as compared to WT mice (Figure 2B). In BM, there was a mean 18.1 fold and 50 fold increase in SHIP^−/−^ and SHIP^ΔIP/ΔIP^ as compared to their WT littermates, respectively. The absolute number of splenic MKP was increased by more than 30 fold in both strains of mice. SHIP^−/−^ PB contained fewer MKP (3 to 5 MKP/µl), but MKP numbers were significantly higher in SHIP^ΔIP/ΔIP^ mice compared to control mice (Figure 2B). We then combined the number of MKP present in the BM and spleen in SHIP deficient mice to establish the total MKP body number. In both mice strains (SHIP^−/−^ and SHIP^ΔIP/ΔIP^), the total MKP pool was increased by a mean of 3.5 fold as compared to their respective WT littermates (Figure 2C).

To further evaluate the functional capacity of MK precursors (MKP) in SHIP deficient hematopoietic organs, we determined CFU-MK activity in both the spleen and BM. We observed an increased CFU-MK content in SHIP deficient BM although the difference did not reach statistical significance (p>0.05). CFU-MK in SHIP deficient spleen were statistically significantly increased (p<0.05) compared to WT, corroborating flow cytometry findings (Figure 2D).

### SHIP deficient MK are redistributed within hematopoietic organs

**Figure 3:**
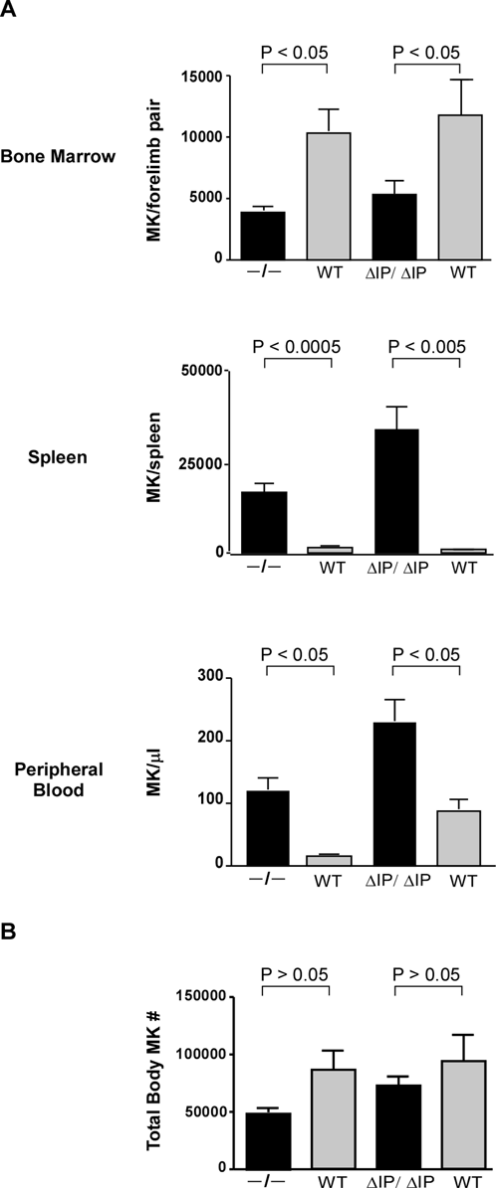
SHIP deficient mice MK are redistributed within hematopoietic organs. (A) Represented are the mean±SEM (n = 4 for experimental groups WT C57Bl6 and SHIP^−/−^, while n = 6 for experimental groups WT 129SvJ and SHIP^ΔIP/ΔIP^) of the absolute numbers of MK (Lin^−^c-Kit^−^CD41^+^) in BM, spleen and PB of SHIP deficient mice (black columns) compared to respective WT littermates (gray columns). Mice strains specified in the graphs. (B) Represented is the mean±SEM (n = 4 for experimental groups WT C57Bl6 and SHIP^−/−^, while n = 6 for experimental groups WT 129SvJ and SHIP^ΔIP/ΔIP^) of total body numbers of MK determined as the calculated sum of MK numbers in one whole spleen plus MK in one femur ×16.6 (since one femur is estimated to contain 6% of the total marrow) [3]. Significance was established using the Mann-Whitney test. *p* values indicated on each graph.

Despite an increase in total MKP numbers in both strains of SHIP deficient mice, BM MK content was decreased by a mean 2 fold in SHIP^−/−^ and SHIP^ΔIP/ΔIP^ while the absolute number of splenic MK was increased by a mean 10.9 fold in SHIP^−/−^ spleen and an even more dramatic increase in SHIP^ΔIP/ΔIP^ spleen. Furthermore, SHIP^−/−^ PB showed a mean 7.7 fold increase in the absolute number of MK and the same trend was observed for SHIP^ΔIP/ΔIP^ mice (Figure 3A). Nevertheless the total MK content in SHIP deficient mice was equivalent to WT littermates (Figure 3B). These observations suggest a shift in the site of megakaryocytopoiesis from the BM to the spleen. This is in agreement with other findings suggesting that the spleen of SHIP deficient mice becomes a site of intense extramedullar hematopoiesis [3], [6], [8].

### SHIP deficient MK have reduced CXCR-4 surface expression

**Figure 4:**
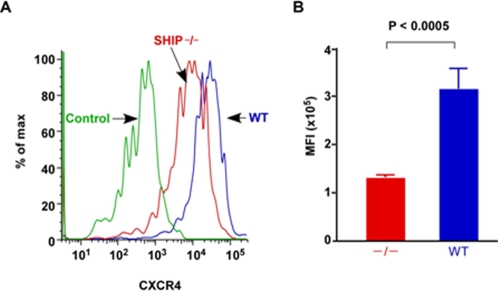
SHIP^−/−^ MK have lowered CXCR-4 receptor expression. (A) Histogram representing the level of CXCR-4 receptor expression on SHIP^−/−^ (black line) and WT (gray line) Lin^−^c-kit^−^CD41^+^ cells in BM. Isotype antibody control indicated in the histogram. (B) Represented is the mean±SEM of the mean fluorescence (MFI) intensity of CXCR-4 expression on SHIP^−/−^ (−/−) (n = 4) and WT (n = 4) Lin^−^c-kit^−^CD41^+^ in BM. Significance was established using the Mann-Whitney test. *p* values indicated in each graph.

We identified that SHIP^−/−^ HSC have fewer CXCR-4 receptors indicating that SHIP deficiency may also compromise SDF-1/CXCR-4 signaling in hematopoietic cells [8]. CXCR-4^+^ cells mobilize towards an increased SDF-1 gradient determining their localization to the BM, the organ with highest SDF-1 concentration [14], [16], [29]. In order to address whether increased MK content in spleen is due to migration between organs we evaluated CXCR-4 expression in SHIP deficient MK. Flow cytometry analysis revealed that BM SHIP^−/−^ MK exhibit a significant reduction in CXCR-4 expression as compared to WT MK (Figure 4A). The mean fluorescence intensity (MFI) for CXCR-4 on SHIP^−/−^ MK was 1.29±0.06 ×10^4^, while it was 3.12±0.45 ×10^4^ in WT littermates (Figure 4B). This mean 2.4 fold decrease in CXCR-4 expression in BM SHIP^−/−^ MK may influence the ability of these cells to home and/or be retained in the BM.

### Phosphorylation of signaling proteins in SHIP deficient MKP

**Figure 5:**
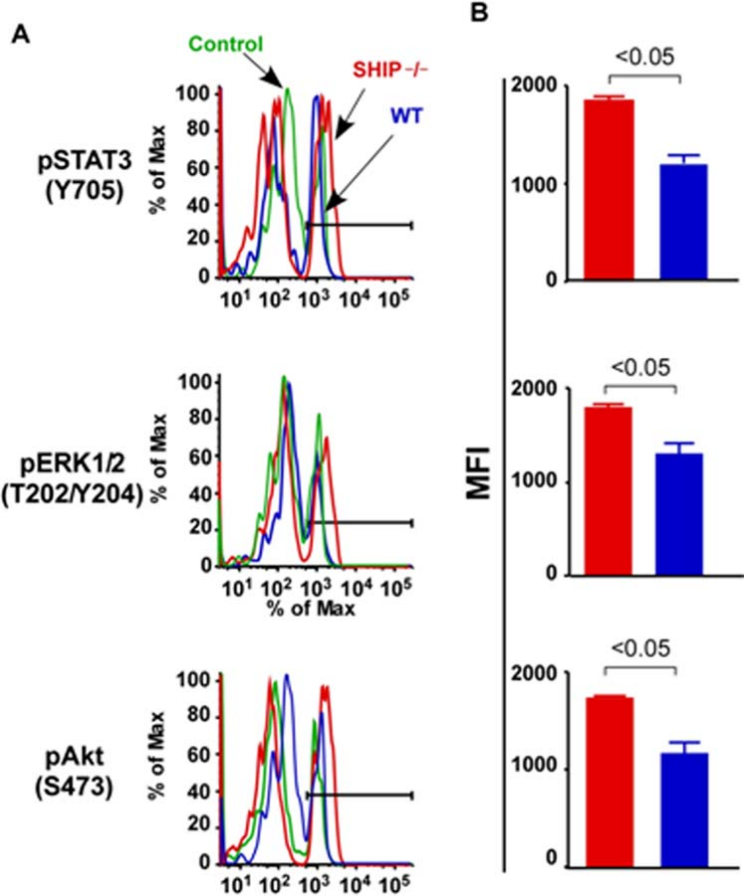
Increased phosphorylation of signaling proteins in SHIP^−/−^ MKP in BM. (A) Histogram representing the level of phospho-STAT3, phospho-Erk1/2 and phospho-Akt in Lin^−^c-kit^+^CD41^+^ BM cells isolated from SHIP^−/−^ (black line) and WT (gray line) mice. Isotype antibody control indicated on the histogram. (B) Represented is the mean±SEM of the mean fluorescence (MFI) intensity of phospho-STAT3, phospho-Erk1/2 and phospho-Akt on SHIP^−/−^ (−/−) (n = 4) and WT (n = 4) Lin^−^c-kit^+^CD41^+^ in BM. Significance was established using the Mann-Whitney test. *p* values indicated on each graph.

SHIP is known to negatively influence signaling proteins downstream of cytokine receptors involved in MK differentiation and/or development; therefore we assessed phosphorylation of the major signaling proteins in SHIP deficient MKP. We observed as expected that SHIP^ΔIP/ΔIP^ BM MKP exhibit enhanced STAT-3, Erk1/2 and Akt phosphorylation (P) when compared to WT as determined by flow cytometry (Figure 5A). SHIP^ΔIP/ΔIP^ MKP exhibited an average 1.5 fold increased in MFI values for P-STAT3, P-Erk, and P-Akt (Figure 5B). Interestingly, there were no significant differences in the percentage of cells containing P-Stat3, P-Erk1/2 and P-Akt (data not shown) between SHIP^ΔIP/ΔIP^ MKP and WT MKP, but rather a difference in the level of phosphorylation on a per cell basis.

### SHIP deficient MK precursors preserve endomitosis function

**Figure 6:**
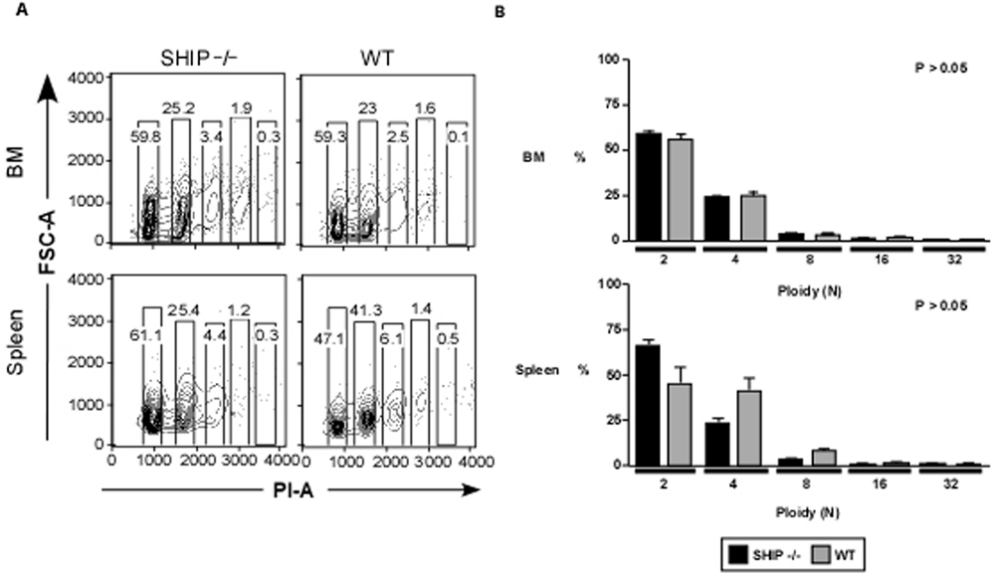
SHIP deficient MK precursors preserve endomitotic function. BM and splenocytes from SHIP^−/−^ and WT mice were enriched for CD41^+^ on AutoMACS, stained with Lin-panel and CD41-biotin/SA-APC, fixed/permeated and stained with PI in the presence of RNase. (A) Representative density plots of ploidy distribution of megakaryocytic cells. Lin^−^CD41^+^ cells were first selected by gating forward and side scatter by area and width. Propidium iodide (*x* axis) versus forward side scatter (*y* axis) density plots were then generated to assess ploidy distribution in BM (top panels) and spleen (bottom panels) of SHIP^−/−^ and WT mice. (B) Represented is the mean±SEM (n = 4 for each experimental group) percentage of BM (top) and spleen (bottom) Lin^−^CD41^+^ cells in different ploidy stages from 2N to 32 N. Statistical analysis was done using the Mann-Whitney test. *p* values indicated on each graph.

Megakaryocytic precursors, unlike other cells, continue to synthesize DNA during differentiation. We next evaluated the ploidy distribution of SHIP^−/−^ Lin^−^CD41^+^ selected cells that morphologically appeared to have fewer nuclei lobules (micro-MK) compared to hyperlobulated MK in WT BM as shown in Figure 1. DNA content of Lin^−^CD41^+^ cells was determined by flow cytometry on BM cells and splenocytes from SHIP^−/−^ and WT mice, after enriching for CD41^+^ cells and staining with propidium iodide (Figure 6A). Ploidy distribution in SHIP deficient mice was similar to wild type littermates (p>0.05), thus, having a comparable ability to mature properly as endomitosis is an obligatory step towards differentiation (Figure 6B).

### SHIP deficient mice have diminished circulating platelets and red blood cells

Since MK numbers are not increased in SHIP deficient mice despite the increased MKP pool, we next evaluated whether terminal MK differentiation into circulating platelets was preserved in these mice. Analysis of SHIP^−/−^ and SHIP^ΔIP/ΔIP^ PB by hematolizer revealed that these mice have a statistically significant reduction in their platelet numbers as compared to WT littermates (Table 1). Furthermore, both strains of SHIP mutant mice have a significantly lower hematocrit percentage compared to WT littermates (Table 1). It remains to be determined whether thrombocytopenia could be related to hypersplenism due to significant increased spleen size in SHIP deficient mice [3], [6], [8].

**Table 1:** Platelet and Hematocrit counts in SHIP-deficient mice.

Mice genotype	Platelet levels (#×10^3^/µl)	Hematocrit (%)
SHIP^−/−^ (n = 6)	672.8±43.4†	42.5±1.4*
SHIP^+/−^ (n = 10)	848.9±35.7	45.2±0.5
SHIP^+/+^ (C57Bl6) (n = 12)	803.7±31.5	46.6±0.5
SHIP^ΔIP/ΔIP^(n = 5)	455.4±81.1†	43.1±1.4†
SHIP^+/ΔIP^(n = 9)	652.3±28.0	46.8±0.5
SHIP^+/+^(129SvJ)(n = 8)	647.9±28.0	47.0±0.6

* p>0.05 compared to their respective WT littermates.

† p<0.05 compared to their respective WT and SHIP heterozygous littermates.

## Discussion

Using two strains of SHIP mutant mice with either deletion of the SHIP promoter/first exon (SHIP^−/−^ mice) [7] or the inositol phosphatase domain (SHIP^ΔIP/ΔIP^) [35], we report that ablation of SHIP results in an increased number of early megakaryocyte progenitors (Lin^−^c-Kit^+^CD41^+^-MKP) in BM, spleen and PB of SHIP deficient mice as determined by flow cytometry and functional assay (CFU-MK). SHIP deficient MKP showed enhanced activation of STAT-3, AKT and ERK

SHIP deficient MKP altered phenotype could be related to unknown intrinsic mechanism and/or related to the cytokine profile of these mice. SHIP deficient mice are characterized by an inflammatory state associated with increased numbers of inflammatory cells [6]. SHIP deficient mice have been shown to have increased levels of IL-6 [5] and TPO (Kerr et al, unpublished results) that could contribute to enhance megakaryocytopoiesis in these mice.

In contrast, the total number of differentiated megakaryocytes (Lin^−^c-Kit^−^CD41^+^ MK) was unaltered in SHIP deficient mice. Furthermore, DNA endoduplication was not affected in SHIP deficient megakaryocytes. However, MK were predominately localized to the spleen and blood with decreased numbers in the BM. We postulate that aberrant MK localization could be related to extramedullary hematopoiesis of SHIP deficient mice since they have increased HSC content in the spleen [3], [8] and/or related to MK migration. We have shown that MK (Lin^−^c-Kit^−^CD41^+^) have decreased CXCR-4 expression that could potentially contribute to their spleen localization instead of retention to the BM space given the higher SDF-1 concentration in this organ.

SHIP deficient mice also exhibit decreased number of platelets as previously shown [31]. In addition, both strains of SHIP deficient mice exhibit a decrease in hematocrit percentages as previously shown in F6-7 SHIP^−/−^ mice [31]. This finding suggests that SHIP may be involved in modulating signaling pathways of early precursors involved in both the MK and erythroid lineages.

In conclusion, our results suggest that SHIP negatively controls pathways promoting early megakaryocytopoiesis without compromising mechanism underlying megakaryocytic endomitosis. Increased MKP compartment without a proportional increase of more differentiated MK and platelets in SHIP deficient mice may support the hypothesis that early megakaryocytopoiesis and late megakaryocyte development and platelet shedding may be regulated by distinct mechanisms.

## Materials and Methods

### Mice strains

SHIP^−/−^ mice (F9 or F10×C57BL6/J) produced in our laboratory have a deletion of the SHIP promoter and first exon [7]. A second SHIP deficient mouse model was also analyzed, SHIP^ΔIP/ΔIP^ (129SvJ) [36], in which the inositol phosphatase domain is deleted (kindly provided by Dr. Jeffrey Ravetch, Rockefeller University, NY, USA). All studies described herein were conducted on six to eight week-old adult mice. Experiments were performed in compliance with institutional guidelines of the University of South Florida.

### Cell isolation

Isolation of BM cells and splenocytes was as described previously [8]. Following red blood cell (RBC) lysis, the cells were resuspended in staining media [8]. PB was obtained from the retro-orbital sinus, sub-mandibular, or heart. For MKP analysis of PB, RBC were lysed twice in 1× RBC lysis buffer (eBioscience). Cells were then resuspended for antibody staining.

### Flow cytometry analysis and antibodies

Staining of MKP and MK was performed as per Hodohara et al [34]. All antibodies were from BD Biosciences except when mentioned. The cells were treated with anti-CD16/CD32 (2.4G2) to block Fc receptors and then stained with a Lin panel-PE, CD41-FITC (MWReg30), and c-Kit-APC (2B8). The Lin- panel was CD3ε (17A2), CD4 (GK1.5), CD8α (53-6.7), B220 (RA3-6B2), Gr-1 (RB6-8C5), Mac-1 (M1/70) (Caltag, Burlingame, CA) and Ter119 (TER-119). Dead cells were excluded using 7-AAD (BD Biosciences). Data was acquired on a FACS Calibur using the FACS Quest software and analysis was done using FlowJo 4.5. For the CXCR-4 analysis, the antibodies mentioned above were used to define the MKP population. CXCR-4 antibody (2B11/CXCR-4) conjugated to biotin was added to the stain, which was revealed using a streptavidin-PeCy7 antibody. For this stain, dead cells were excluded using DAPI (Sigma-Aldrich) and data was acquired on a FACS LSR (BD Dickenson).

### Platelet analysis

Blood was collected by heart puncture using a 25 gauge needle with an EDTA coated 1 ml syringe, or by sub-mandibular bleed. For each method the blood was collected directly into microtainer K_2_EDTA tubes (BD, Franklin, NJ, USA). 200 µl of blood (either diluted 1∶4 or whole blood) was then analyzed on the CellDyn 3700 hematology analyzer using the Veterinary Package Software (Abbott Diagnostic, Dallas, Texas USA), with a ratio to default corresponding to the mice strain studied.

### Histopathology

Bone and spleen were fixed in 10% buffered paraformaldehyde. Bones were first decalcified using Routine Acid Decalcification (RDO; Apex engineering Products, Plainfield, IL, USA), followed by sectioning. The spleen and bone sections were then stained with Hematoxylin-Eosin after being deparaffinized with xylene or stained with Glycoprotein IIB-IIIA (CD41) antibody. Briefly, slides were stained using the modified Meyer's Hematoxylin and Eosin phloxine B solution (Fisher Scientific, Suwanee, GA, USA) according to a modified Armed Forces Institute of Pathology (AFIP) protocol. Cells were visualized on a Leica DM LB microscope. Pictures were taken using a RTcolor Spot camera (Diagnostic Instrument Inc) with Spot Advance v3.0 software.

### Ploidy assay

BM cells were isolated from hind/fore limb and vertebral column. Briefly, cells were incubated on ice with CD41-biotin antibody (Serotec, Raleigh, NC, USA) and then with anti-biotin beads (Miltenyi Biotec) at 4°C. CD41^+^ cells were then selected using the autoMACS (Miltenyi Biotec). Collected CD41^+^ cells were stained with Lin panel-FITC as mentioned above and with streptavidin-APC (BD Biosciences). The cells were fixed in 1% formaldehyde (Sigma-Aldrich) for 15 minutes on ice and then incubated overnight in a 70% ethanol solution at −20°C. The day after, the cells were washed carefully of any residual ethanol with PBS and incubated for at least 3 hours in a propidium iodine (PI) solution composed of 50 µg/ml PI, 0.1% Triton X-100, 2.5 U/ml RNase in PBS without Ca or MG (all from Sigma-Aldrich except for the PBS which was from Gibco BRL/Invitrogen).

### CFU-MK assay

Hundred thousand 10^5^ whole BM cells were assessed for megakaryocyte progenitor activity in a CFU-Mk assay, MegaCult-C (Stem cell Technologies, Vancouver, CA) per the manufacturer's instructions.

### Phosphorylation analysis of signaling proteins

Phospho-flow analysis was performed on BM cells isolated from 129SvJ WT and SHIP^ΔIP/ΔIP^. Cells were isolated on ice, after red blood cell lysis, cells were stained for Lin^−^c-kit^+^CD41^+^ using the same antibody clones than mentioned above, except for c-kit which was conjugated to PeCy7 instead of APC. Cells were then directly fixed with Fix buffer (BD Biosciences) for 10 minutes at 37°C and washed with Perm/Wash buffer I (BD Biosciences). After centrifugation, the cells were resuspended in Perm/Wash buffer I. The appropriate phospho-antibody was added to the cells, and incubated at RT for 30 minutes. The cells were then washed twice in Perm/wash buffer I and stored in that same buffer on ice until the time of acquisition The phospho-antibodies were from BD Biosciences, anti-phospho-Stat3Y705 (clone 4/P-Stat3), anti-phospho ERK1/2 T202/T204 (clone 20a), anti phosphor Akt S473) (clone F29-763). The analysis was carried on a BD LSR II (BD Biosciences).

### Statistical analysis

Statistical analysis for each experiment consisted of the Mann-Whitney test performed using the Prism software. For each experiment, the number of individual mice analyzed is indicated as “n” in the figure legends. All mice were analyzed individually (i.e. the cells from different animals were not pooled together for these experiments).
